# Development and tracking of inferior vena cava diameter after filter placement using a circle-fitting predictive model

**DOI:** 10.1016/j.isci.2025.113616

**Published:** 2025-09-21

**Authors:** Maofeng Gong, Rui Jiang, Xu He, Jianping Gu

**Affiliations:** 1 Department of Interventional and Vascular Radiology, Nanjing First Hospital, Nanjing Medical University, Nanjing, Jiangsu 210006, P.R. China

**Keywords:** Surgery

## Abstract

The diameter of the inferior vena cava (IVC) is a critical factor in the decision-making process for IVC filter (IVCF) placement. A mismatched filter can compromise its stability and potentially lead to adverse events. This study develops and validates a predictive model using a circle-fitting algorithm to estimate the IVC diameter after filter placement. The findings suggest that single-angle measurements may misrepresent the filter-vessel compatibility, since the IVC transforms from an oval to a near-circular cross-section following filter placement. The predictive model, integrating three-dimensional, multi-angle cavography with circumference-based calculations, demonstrates excellent concordance with both post-placement maximum and minimum diameters in animal and clinical samples. Compared to the conventional single-angle measurements prior to IVCF placement, the predictive diameter can provide a more accurate and reliable representation of the IVC size, which was superior to either the maximum or minimum diameter alone, suggesting a higher predictive reliability. This model may provide a valuable tool for more accurate IVCF diameter selection and support future research.

## Highlights

• Proposes a predictive model with circle-fitting algorithm to estimate IVC diameter
• Predictive diameter matches post-placement IVC diameter, with circular shape shift
• Model provides more accurate IVC size estimation than single-angle measurements
• Superior to maximum or minimum diameter alone, with higher predictive reliability

## Graphical abstract

**Figure:**
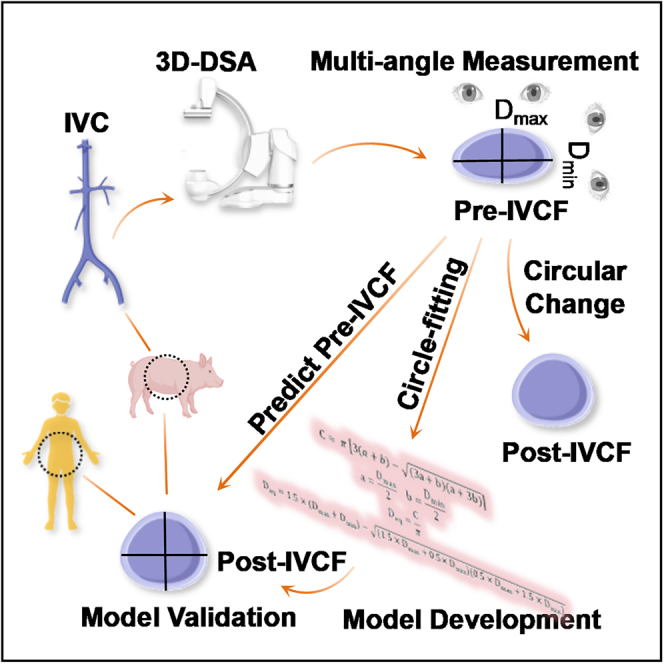

## Introduction

The use of inferior vena cava (IVC) filters (IVCFs) remains a common strategy for preventing potentially fatal pulmonary embolism in selected patients with deep vein thrombosis.^1^ However, their clinical utilization and overall benefits are still a subject of ongoing debates due to the associated risk of adverse events, such as migration, fractures, tilt, IVCF-related thrombosis, and IVC wall penetration.^2^^,^^3^ IVCF migration is not uncommon and may trigger off catastrophic consequences, particularly in patients with an IVC diameter of ≥28 mm.^4^ Conversely, a smaller IVC diameter has been linked to an increased risk of intimal hyperplasia (IH) and collagen fiber proliferation,^5^ which consequently reduces the successful retrieval rate of IVCFs.^6^ Accurate assessment of IVC diameter prior to IVCF placement is, therefore, essential for mitigating these risks.

Over the years, studies have sought to identify the potential factors that contribute to IVCF-related complications, with the goal of minimizing risks and improving the long-term outcomes.^3^^,^^5^^,^^6^^,^^7^ One key mechanism underlying filter-related complications is the mismatch between IVCF and IVC diameters, which alters local biomechanical forces after placement.^5^^,^^8^^,^^9^^,^^10^ Spindle-shaped IVCFs, although associated with a lower risk of IVC perforation than umbrella-shaped designs,^11^ are currently available in only a few diameters, limiting individualized choice for patients with varying IVC diameters. In contrast, arterial stents have a well-established diameter-matching system that helps reduce the incidence of migration and perforation. The absence of standardized sizing principles for IVCFs means that optimal selection for different IVC diameters is often not possible in clinical practice.

Geometric and mechanical mismatches may alter the contact area and biomechanical forces distribution between the IVCF and IVC wall: excessive IVCF diameter or force can disrupt local hemodynamics and vessel wall stress, promoting IH and thrombosis,^5^^,^^8^^,^^12^ whereas undersized IVCF or insufficient fixation increase the risk of migration.^4^ Therefore, single-diameter IVCFs cannot meet the anatomical needs of all patients, highlighting the urgent need for multi-diameter IVCF and standardized selection criteria to reduce complication rates. However, the quantitative relationship between this mismatch and complication risk remains unclear, partly due to the lack of accurate IVC diameter measurement methods and insufficient understanding of post-placement morphological changes. Developing reliable IVC diameter measurement and prediction methods, and integrating them to filter design and clinical decision making, is thus a critical clinical challenge.

Although several modalities are used for IVC diameter assessment, the standardized measurement is lacking, and accuracy as well as clinical practicality remain suboptimal. An alternative modality, such as cavography based on digital subtraction angiography (DSA), is often utilized to evaluate anatomical variants prior to IVCF placement.^6^^,^^13^^,^^14^ The IVC diameter is typically measured using these two-dimensional images. However, there remains no consensus on which imaging plane, coronal or sagittal, should be referenced when evaluating the IVC diameter. Moreover, the monography of the IVC, including geometry and orientation, may shift from an oblique orientation to a more circular geometry following IVCF placement.^12^^,^^15^^,^^16^ Accordingly, Xiao et al. suggested that a circumference-based calculated diameter derived from computed tomography (CT) may be more appropriate for evaluating the risk of migration.^15^ However, this approach increases the reliance on additional cross-sectional CT imaging, potentially raising both radiation exposure and healthcare costs. Therefore, there is a need for a predictive model based on two-dimensional DSA image that could accurately predict the post-placement IVC diameter, providing a more practical and cost-effective clinical application.^5^^,^^8^ Such a model may provide theoretical and methodological basis for optimizing IVCF diameter design by more precisely measuring and predicting changes in the IVC diameter, thereby reducing the risk of IVCF-related clinical complications. Despite this, no such model has yet been established.

In this work, based on a circle-fitting algorithm, along with experimental and clinical data, we aim to develop and validate a predictive model based on DSA device to estimate the IVC diameter following IVCF placement, thereby providing a more accurate risk stratification tool for IVCF-related complications.

## Results

### Baseline characteristics and IVC diameter feathers

A total of 24 healthy Bama miniature swine (12 males and 12 females, aged 45–55 weeks, body weight 25–35 kg) were included in this study. Technical success of IVCF placement was achieved in all animals (24/24).

**Figure 1:**
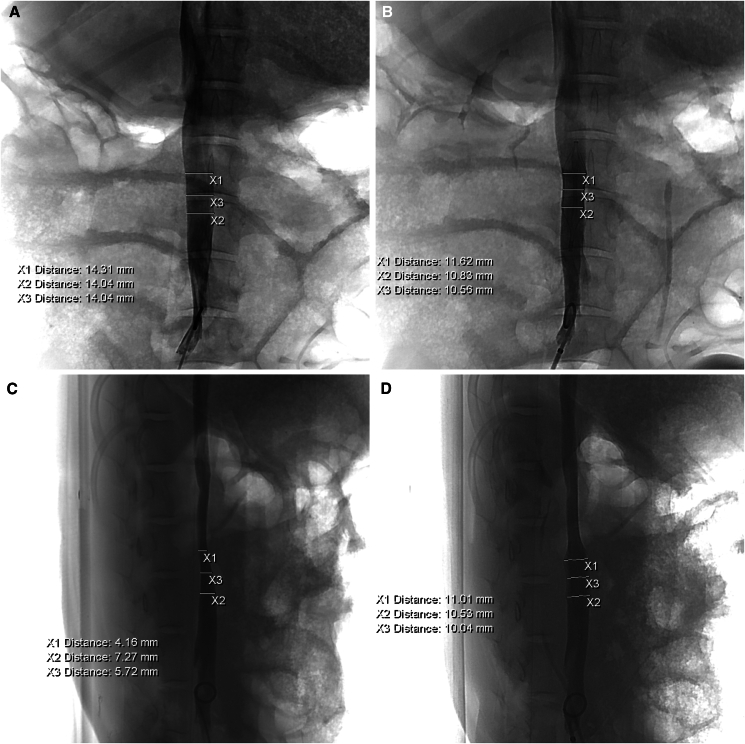
Diameter measurements before and after IVCF placement (A) Measurement of IVC diameter at the optimal fluoroscopic angle for D_max_ prior to filter placement. (B) Measurement of IVC diameter after filter placement. The diameters at the upper (X_1_) and lower (X_2_) edges of the IVCF strut junction were measured, and their average was calculated as D_max-IVCF_. (C) At the optimal angle for D_min_. (D) The same measurements (X_1_ and X_2_) were performed, and the average value was calculated as D_min-IVCF_.

Diameter measurements before and after IVCF placement were illustrated in Figure 1. Cavography of the IVC before IVCF placement showed an oval shape at the intended IVCF placement site, with a mean maximum diameter before filter placement (D_max_) of 15.52 ± 2.82 mm (range, 11.11–21.67 mm) and a mean minimum diameter before filter placement (D_min_) of 8.88 ± 2.01 mm (range, 6.57–13.55 mm), as determined under the guidance of 3D-DSA imaging and multi-angle cavography. The D_max_ was significantly larger than D_min_ (*p* < 0.05); however, no significant differences in D_max_ (*p* = 0.127) or D_min_ (*p* = 0.648) were observed between the Φ32-mm and Φ20-mm filter groups. Notably, the mean predictive model diameter (D_eq_), calculated based on the predictive model, was 12.48 ± 1.81 mm (as shown in Table 1).

**Table 1:** Inferior vena cava diameters before and after placement of Φ32-mm and Φ20-mm filters

Variables	Overall (*n* = 24)	Φ32 mm (*n* = 12)	Φ20 mm (*n* = 12)	*p* value
D_max_, mm, $\bar{x}$ ± SD	15.52 ± 2.82	16.40 ± 2.80	14.63 ± 2.66	0.127
D_min_, mm, $\bar{x}$ ± SD	8.88 ± 2.01	8.71 ± 1.76	9.06 ± 2.31	0.684
D_eq_, mm, $\bar{x}$ ± SD	12.48 ± 1.81	12.90 ± 1.67	12.06 ± 1.91	0.264
D_max-IVCF_, mm, $\bar{x}$ ± SD	12.55 ± 1.77	13.01 ± 1.62	12.09 ± 1.85	0.211
D_min-IVCF_, mm, $\bar{x}$ ± SD	12.59 ± 1.82	13.18 ± 1.64	11.99 ± 1.86	0.109

Abbreviations: D_max_ = maximum diameter before filter placement; D_min_ = minimum diameter before filter placement; D_eq_ = predictive diameter in model; D_max-IVCF_ = maximum diameter after filter placement, D_min-IVCF_ = minimum diameter after filter placement; SD = standard deviation

### Diameter measurement after IVCF placement and validation of the predictive model

**Figure 2:**
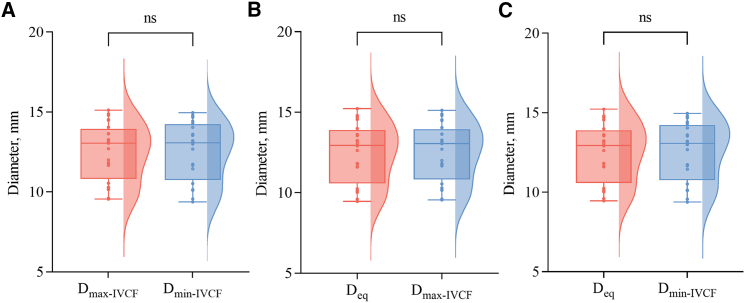
Cloud-rain plots used to visualize the differences among D_eq_, D_max-IVCF_, and D_min-IVCF_ There were no significant differences between D_max-IVCF_ and D_min-IVCF_ (A), D_eq_ and D_max-IVCF_ (B), and D_eq_ and D_min-IVCF_ (C) (all *p* > 0.05).

**Figure 3:**
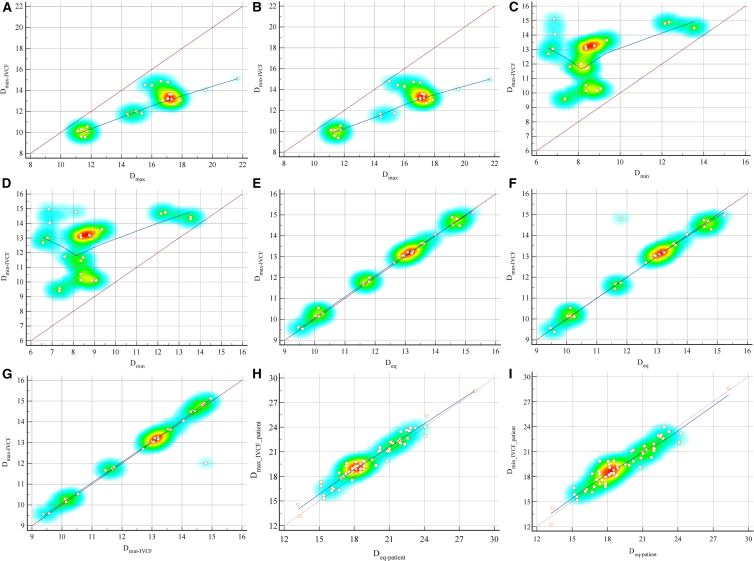
Validation of the predictive circumference-based calculated diameter model (A and B) D_max_ demonstrated fair to good agreements with D_max-IVCF_ (Lin’s CCC = 0.431, 95% CI: 0.251–0.583) and D_min-IVCF_ (CCC = 0.427, 95% CI: 0.238–0.585). (C and D) D_min_ had poor agreements with D_max-IVCF_ (CCC = 0.152, 95% CI: 0.006–0.292) and D_min-IVCF_ (CCC = 0.136, 95% CI: −0.011–0.276). (E and F) In swine, D_eq_ showed excellent agreement with the measured D_max-IVCF_ (CCC = 0.996, 95% CI: 0.991–0.998) and the measured D_min-IVCF_ (CCC = 0.937, 95% CI: 0.860–0.972). (G) Excellent concordance was observed between D_min-IVCF_ and D_max-IVCF_ (CCC = 0.946, 95% CI: 0.878–0.976), indicating that the IVC morphology tended to become circular following IVCF placement. (H and I) In patients, D_eq-patient_ showed excellent agreement with the measured D_max-IVCF-patient_ (CCC = 0.939, 95% CI: 0.904–0.961) and the D_min-IVCF-patient_ (CCC = 0.949, 95% CI: 0.917–0.969).

The mean maximum diameter after filter placement (D_max-IVCF_) was 12.55 ± 1.77 mm, while the mean minimum diameter after filter placement (D_min-IVCF_) was 12.59 ± 1.82 mm, with no statistically significant difference between the two (*p* = 0.943). Statistical comparison was conducted between these measured values, and D_eq_ was derived from the predictive model. No significant differences were found between D_max-IVCF_ (*p* > 0.05) and D_eq_, or between D_min-IVCF_ and D_eq_ (*p* > 0.05), as illustrated in the cloud-rain plots (Figure 2). Lin’s concordance correlation coefficient (CCC) demonstrated excellent agreement between D_max-IVCF_ and D_min-IVCF_ (CCC = 0.946, 95% confidence interval [CI]: 0.878–0.976, Pearson *ρ* = 0.946) (Figure 3), illustrating that IVC underwent morphological adaptation, transforming from an oval to a near-circular configuration following IVCF placement. Similarly, D_eq_ showed excellent agreement with both D_max-IVCF_ (CCC = 0.996, 95% CI: 0.991–0.998, Pearson *ρ* = 0.997) and D_min-IVCF_ (CCC = 0.937, 95% CI: 0.860–0.972, Pearson *ρ* = 0.939). These findings suggested that the D_eq_ derived from the predictive model effectively predicts the IVC diameter post-IVCF, thus validating the model’s precision and reliability.

D_max_ demonstrated fair to good agreements with D_max-IVCF_ (CCC = 0.431, 95% CI: 0.251–0.583, Pearson *ρ* = 0.878) and D_min-IVCF_ (CCC = 0.427, 95% CI: 0.238–0.585, Pearson *ρ* = 0.843), and D_min_ had poor agreements with D_max-IVCF_ (CCC = 0.152, 95% CI: 0.006–0.292, Pearson *ρ* = 0.454) and D_min-IVCF_ (CCC = 0.136, 95% CI: −0.011–0.276, Pearson *ρ* = 0.401). Among the three parameters, D_eq_ showed the highest predictive accuracy. Specifically, D_eq_ was superior to both D_max_ (95% CI:0.967–0.997, *p* < 0.001) and D_min_ (95% CI: 0.982–0.998, *p* < 0.001) in predicting D_max-IVCF_. Likewise, D_eq_ was better in predicting D_min-IVCF_ when compared to D_max_ (95% CI: 0.573–0.953, *p* < 0.001) and D_min_ (95% CI: 0.749–0.975, *p* < 0.001). Moreover, the predictive ability of D_eq_ for the D_max-IVCF_ is superior to that for D_min-IVCF_ (95% CI: 0.658–0.964, *p* < 0.001).

### Model validation in patient samples

Among the 62 eligible patients included, the mean age was 57.7 ± 18.2 years, with 51.6% (32/62) being male. The IVCF used were OptEase (44/62) and Aegisy (18/62). The mean D_max-patient_ and D_min-patient_ were 22.07 ± 3.99 mm and 15.95 ± 3.99 mm, while the D_eq-patient_ was 19.24 ± 2.88 mm. Following the IVCF placement, the mean D_max-IVCF-patient_ and D_min-IVCF-patient_ were 19.93 ± 2.76 mm and 19.34 ± 2.71 mm, respectively, with no statistically significant differences found (*p* = 0.237). Additionally, no significant differences were found between D_max-IVCF-patient_ and D_eq-patient_, or between D_min-IVCF-patient_ and D_eq-patient_ (both *p* > 0.05). Lin’s CCC demonstrated excellent agreement between D_eq-patient_ and D_max-IVCF-patient_ (CCC = 0.939, 95% CI: 0.904–0.961, Pearson *ρ* = 0.968), as well as between D_min-IVCF-patient_ (CCC = 0.949, 95% CI: 0.917–0.969, Pearson *ρ* = 0.951).

## Discussion

In this experimental study, a circle-fitting algorithm, integrating 3D-DSA, multi-angle cavography, and optional angle cavography with circumference-based calculations, was used to develop the predictive model for estimating IVC diameter following IVC placement. This predictive diameter showed an excellent agreement between post-placement maximum and minimum diameters, confirming the IVC’s morphological adaptations toward a more near-circular configuration. Compared to the conventional single-angle measurements prior to IVCF placement, the predictive diameter can provide a more accurate and reliable representation of the IVC size, which was superior to either the maximum or minimum diameter alone, suggesting a higher predictive reliability.

IVC diameter should be considered in the decision to use an IVCF.^9^ The placement of a mismatched IVCF may have an impact on the stability of implanted IVCF and lead to significant adverse outcomes, such as migration, IVC perforation, thrombosis, or IH.^5^^,^^8^^,^^11^^,^^15^^,^^16^ Given the critical importance of accurate IVC diameter measurement in ensuring appropriate IVCF selection, several methods have been proposed.^13^^,^^15^ Among them, the circumference-based calculation method appears particularly promising, as it provides a more accurate assessment by accounting for the morphological transformation of IVC from an oval to a near-circular configuration following IVCF placement.^15^^,^^16^ Although pre-procedural CT offers substantial added values in the precise measurement of IVC diameter, no clinical practice guidelines to date recommend its routine use for IVCF placement.^12^ Instead, DSA-guided cavography remains a common technique for the anatomical and positional assessment prior to IVCF placement.^9^^,^^13^ While DSA-based measurements have certain limitations, they have the following potential advantages: (1) real-time visualization, (2) minimal additional radiation exposure or scheduling delays, (3) immediate diagnosis and intervention, and (4) simplicity and widespread availability. Therefore, the present study established a predictive model for IVC diameter estimation integrating 3D reconstruction with two-dimensional cavography.

Based on cross-sectional CT imaging, Xiao et al.^15^ reported that IVC morphology varies among individuals and can be categorized into five types according to geometric characteristics and orientation: Type I, an oval shape with a left anterior oblique orientation relative to the horizontal line (66.5%); Type II, a circular shape (3.3%); Type III, a vertically oriented oval (0.5%); Type IV, a horizontally oriented oval (5.0%); and Type V, an irregular shape (24.7%). Thus, oval IVC shapes are most common (72%–78.8%), while circular shapes are relatively rare (3.3%–3.5%).^6^^,^^15^ Given these variations of shape, comprehensive imaging evaluations of IVC prior to IVCF placement are of great importance. This study indicated that cavography from a single projection angle may introduce measurement bias when assessing the true diameter, thereby compromising the accuracy of filter-to-vessel matching. Our experimental results similarly confirmed the predominance of oval IVC morphology in swine. The D_max_ and D_min_ were obtained using 3D-DSA and multi-angle cavography, selecting the optimal projection angle to capture the maximum and minimum diameters. A predictive model was developed based on a circle-fitting algorithm, incorporating the parameters of minimum and maximum diameters using mathematical formula. This model demonstrated excellent predictive performance for the post-procedure maximum diameter (CCC = 0.996) and minimum diameter (CCC = 0.937), which were also validated in patients on cross-sectional CT venography (CTV) with maximum diameter (CCC = 0.939) and minimum diameter (CCC = 0.949). These findings may have some clinical implications, suggesting that an appropriate-size IVCF can be chosen based on the predicted IVC diameter, derived from baseline pre-filter insertion measurements, which appeared to be similar to the actual IVC diameter measured after placement. This enhanced accuracy facilitates personalized and optimal IVCF sizing, potentially reducing filter-related adverse events and improving retrieval success rates.

Previous studies analyzed changes in IVC morphology and diameter before and after IVCF placement using CTV.^15^^,^^16^ The findings demonstrated a more pronounced anisotropic dimensional changes in response to IVCF placement, with the IVC tending toward a near-circular shape following IVCF procedure. Consistent with these clinical findings, the present swine model study observed substantial alterations in both D_max_ and D_min_. A strong concordance between D_max-IVCF_ and D_min-IVCF_ in swine and patients further confirmed the circularization tendency of the IVC post-procedure. These alternations suggested that the IVC cannot collapse as much as it did prior to IVCF placement, which had some important implications. There is increasing risk of the IVCF becoming adherent over time, as the IVCF struts may become embedded into the IVC wall with each respiratory cycle, making removal difficult and potentially necessitating surgical extraction.^17^ Moreover, the absence of cycling changes in diameter during respiration may impede venous return flow, potentially inducing lower limb or IVCF thrombosis.^10^^,^^18^

Based on 3D-DSA, multi-angle cavography, and optimal-angle cavography, the agreement between the predicted and actual measurements were assessed and demonstrated the proposed algorithm performs very close to the manual measurements. These findings suggested that integrating both D_max_ and D_min_ yields more accurate predictions of post-procedural diameter than single-angle measurement alone (*p* < 0.001). The predictive diameter derived from this model tends to reliably predict the diameter changes following IVCF placement. These findings may generate clinical implications by dominating preoperative IVCF selection and identifying appropriate IVC segment to minimize the post-procedural IVCF complications.

This study may provide some clinical implications by providing a reliable predictive model for estimating the IVC diameter following IVCF placement, addressing a key challenge in clinical practice. Theoretically, the diameter of the IVCF should be slightly larger than the IVC diameter to maintain an appropriate oversizing ratio, ensuring stable fixation of the filter and reducing the risk of migration. However, an excessively small IVC diameter may trigger off excessive stimulation to the venous wall, thereby exacerbating IH, which is one of the main causes of difficult or failed filter retrieval.^5^^,^^6^^,^^8^ The predictive model established in this study may provide a theoretical basis for more accurately assessing the close correlations between IVCF diameter and IVCF migration, as well as IVC IH. Moreover, the validation in both animal experiments and human clinical data demonstrated its stability and translational potential. It could be integrated into procedural imaging platforms and is available for prospective testing. In clinical practice, this predictive model could support interventionalists in preoperative planning, guiding tailored further IVCF selection and placement, thereby enhancing procedural long-term outcomes. Ultimately, this advancement contributes to optimizing the design of IVCF diameters to achieve precise matching between the IVCF and IVC and promoting individualized patient care in venous thromboembolism management.

In conclusion, a circle-fitting algorithm was developed for precisely estimating the IVC diameter based on DSA imaging. Since the IVC tends to adopt a near-circular configuration following IVCF placement, the use of a circle-fitting algorithm that integrates 3D multi-angle cavography with circumference-based calculations provides a more accurate assessment of IVC diameter prior to IVCF placement compared to using either the maximum or minimum diameter alone. Our findings suggest that modeling the IVC as a circular configuration after IVCF placement provides a reliable approximation of its actual dimensions and may serve as a potentially practical tool to support clinical decision making and optimize IVCF diameter selection in future practice.

### Limitations of the study

Several limitations should be acknowledged. First, the incidence of IVCF migration was not directly evaluated in this study because the filter diameter was larger than the vessel, as well the primary purpose was to develop a predictive model to estimate the IVC diameter following IVCF placement. Second, measurement accuracy may have been compromised by suboptimal contrast opacification or artifacts introduced by bowel gas, respiratory motion (animals were not intubated), body habitus, or other technical factors. Thus, a certain degree of error inherent to cavographic measurements appears unavoidable. However, unified measurement standards were adopted both pre- and post-procedure, which minimized potential measurement errors to the greatest extent. Third, the relatively small animal sample size and the use of a spindle-shaped IVCF, rather than umbrella-shaped filters,^7^ may have introduced bias and limited the generalizability of the findings. Therefore, larger samples and clinical study are warranted to validate and extend our findings. Despite these limitations, this is the first study to explore the development of a predictive model for IVC diameter changes following IVCF placement.

## Resource availability

### Lead contact

Requests for further information and resources should be directed to and will be fulfilled by the lead contact, Jianping Gu (gujianpingnj@163.com).

### Materials availability

This study did not generate new materials.

### Data and code availability

• All data reported in this paper may be shared by the corresponding author upon reasonable request.
• This paper does not report original code.
• Any additional information required to reanalyze the data reported in this paper is available from the lead contact upon request.

## Acknowledgments

Graphical abstract and Figure S1 were created with BioRender.com. This work was supported by Jiangsu Medical Association Special Fund Project [(SYH-3201140-0088(2023035)], Nanjing Medical Science and Technology Development Project (YKK23116), and Nanjing Medical University Science and Technology Development Fund Project (NMUB20230163).

## Author contributions

Conceptualization, M.G., X.H., and J.G.; data curation, M.G. and R.J.; formal analysis, M.G.; funding acquisition, M.G.; investigation: M.G., R.J., X.H., and J.G.; methodology, M.G., X.H., and J.G.; project administration, J.G.; writing – original draft, M.G., R.J., X.H., and J.G.; writing – review & editing, M.G., R.J., X.H., and J.G.

## Declaration of interests

The authors declare no competing interests.

## STAR★Methods

### Key resources table

**Table:** 

REAGENT or RESOURCE	SOURCE	IDENTIFIER
**Deposited data**		

Raw and analyzed data	This paper	Shared upon request by the lead contact

**Experimental models: Organisms/strains**		

Bama miniature swine (Sus scrofa domestica)	Laboratory animal center of Nanjing First Hospital	N/A
Patients	Clinical center of Nanjing First Hospital	N/A

**Software and algorithms**		

Circle-fitting algorithm	This paper	N/A
Siemens syngo.via Workspace v21	Siemens Healthineers	https://www.siemens-healthineers.com/products-services
IBM SPSS Statistics v22	IBM Corp.	https://www.ibm.com/products/spss-statistics
R v4.2.3	R Project for Statistical Computing	https://www.r-project.org/
GraphPad Prism v9.0	MDF	https://www.graphpad.com/
Biorender	BioRender	https://www.biorender.com
MedCalc v22.009	MedCalc Software Ltd.	https://www.medcalc.org

**Other**		

Illicium® vena cava filter	Visee Medical Instruments Co., Ltd.	http://www.visee.com.cn/
OptEase® vena cava filter	Cordis	https://cordis.com/apac/products/intervene/endovascular/vena-cava-filters/optease-retrievable-vena-cava-filter
Aegisy® vena cava filter	LifeTech scientific Co., Ltd	https://www.lifetechmed.com/product/p2/s2/vena-cava-filter/20221216/aegisy.aspx
Artis zee digital subtraction angiography machine	Siemens Healthineers	https://www.siemens-healthineers.com/products-services
128-slice dual-source CT scanner (SOMATOM definition flash)	Siemens Healthineers	https://www.siemens-healthineers.com/products-services
Anesthesia machine R620-S1-IEC	Rui Wode Life Science and Technology Co., Ltd	https://www.rwdls.com/product-solutions/life-sciences/modeling/anesthesia

### Experimental model and study participant details

Bama miniature swine were obtained from Laboratory Animal Center of our institution. The swine included 12 males and 12 females, aged 45–55 weeks, with body weight of 25–35 kg. Sex was not considered as a variable in this study. All procedures were performed in accordance with the Guidelines for the Care and Use of Laboratory Animals, and the study protocol was approved by the Animal Ethics Committee of Nanjing First Hospital (No. DWSY-23153536). 62 patients who underwent IVCF placement under spontaneous breathing and received contrast-enhanced CT venography (CTV) both before and within two weeks after this procedure were retrospectively reviewed. Eligible patients were those in whom either an IVCF of OptEase (Cordis, Miami, FL) or Aegisy (Life Tech Scientific Co., Ltd., Shenzhen, China) had been placed. The selection of IVCF types and sizes was primarily based on physician discretion. The mean age was 57.7 ± 18.2 years, with 51.6% (32/62) being male. Sex was also not considered as a variable in this study. The patient sample was entirely East Asian, with all participants of Han Chinese ethnicity. This patient study was approved by the Institutional Review Board of Nanjing First Hospital (No. KY20200117-01), with a waiver of written informed consent due to the retrospective design.

### Method details

#### IVCF devices and anesthesia

The purpose of this study was to investigate spindle-shaped filters and provide a theoretical basis for future improvements; therefore, Illicium filters (Illicium, Visee Medical Instruments Co., Ltd, Shandong, China), one of commonly used spindle-shaped filter type, were included in this study. The filter is manufactured from a nickel-titanium alloy through precision laser engraving. It boasts an integrated structure with six longitudinal struts, offering excellent biocompatibility and flexibility.^5^ To estimate the IVC diameter alternation following different IVCF placement, animals were randomly allocated to receive retrievable Illicium IVCFs^5^ with diameters of either 32 mm (*n* = 12) or 20 mm (*n* = 12).

Prior to the interventional procedures, all swine were premedicated via intramuscular injection of ketamine hydrochloride (5 mg/kg, Hengrui Pharmaceuticals Co., Ltd., Jiangsu, China) and atropine sulfate (0.04 mg/kg, Himed Pharmaceutical Co., Ltd., Shanghai, China). Anesthesia was induced with inhaled isoflurane (100 mL/bottle, Rui Wode Life Science and Technology Co., Ltd, Shenzhen, China) administered through face mask using an anesthesia machine (R620-S1-IEC; Rui Wode Life Science and Technology Co., Ltd, Shenzhen, China). General anesthesia was maintained with isoflurane (3–5 mL/min) and oxygen (0.5–0.8 L/min), and continuous physiological monitoring was conducted throughout the procedures. Images were acquired at the end of inspiration (end-inspiratory phase).

#### Measurement of D_max_ and D_min_, procedures, and circle-fitting algorithm

As previously reported,^5^^,^^8^ accurate assessment of the IVC diameter below the renal veins was achieved using three-dimensional (3D) DSA reconstructions coupled with multi-angle cavography prior to IVCF placement. A schematic illustration of this imagine strategy is presented in Figure S1. DSA imaging was reconstructed into 3D images using post-processing workstation (Siemens Workspace, Germany). Based on 3D DSA reconstructions, multiplanar reconstruction techniques were used to analyze the target IVC region from multiple angles to evaluate the maximal and minimal diameters at the IVC implantation site. By dynamically adjusting the C-arm rotation angles, the optimal projection angles presenting the optimal imaging angle for precise measurement of the minimum diameter (D_min_) and maximum diameter (D_max_). At these locked projection angles corresponding to the D_min_ and D_max_, angiographic acquisitions were performed to capture the D_min_ and D_max_ diameters. Under these conditions, cavography prior to IVCF placement was performed using a non-ionic contrast agent (iodixanol, 370 mgI/mL; Bayer Schering Pharma, Germany) at a rate of 5.0 mL/s, with a total volume of 15 mL. IVC diameters were then measured by repeated measurements performed independently by two experienced interventionalists at the region of interest (ROI) that designated for IVCF placement. The ROI was defined as the segment of the IVC between two anatomical reference points, namely, the superior (X_1_) and inferior (X_2_) margins corresponding to the planned site of filter placement. The mean D_max_ and D_min_ were calculated using the following formula (Equation 1):

(Equation 1) $$D_{m\text{ax}}{\text{or}\;D}_{\min}=\frac{X_{1}+X_{2}}{2}$$

where D_max_ or D_min_ is the mean maximum or minimum diameter measured prior to IVCF placement; X_1_ is the superior margin of the planned site of filter placement, and X_2_ is the inferior margin.

Each IVCF was introduced through a dedicated delivery sheath via either the right or left femoral vein and deployed under fluoroscopic guidance below the level of the renal veins at the measured ROI.

Following IVCF placement, the configuration of IVC tended to approximate a more circular shape.^5^^,^^15^^,^^16^ To standardize measurements and facilitate comparative analysis, circle-fitting algorithm, coupled 3D multi-angle cavography with circumference-based calculations, was applied to develop a predictive model. Specifically, the circumference of the oval IVC was first estimated using the Ramanujan formula (Equation 2), and subsequently converted (Equation 3) into an equivalent circular diameter (Equation 4) using the following equations:

(Equation 2) $$C\approx \pi [3\left(a+b\right)-\sqrt{\left(3a+b\right)\left(a+3b\right)}$$

(Equation 3) $$\begin{array}{c} a=\frac{D_{m\text{ax}}}{2}\\ b=\frac{D_{m\text{in}}}{2} \end{array}$$

(Equation 4) $$D_{\text{eq}}=\frac{C}{\pi }$$

Finally, the circumference-based diameter (D_eq_) from this predictive model were simplified as (Equation 5):

(Equation 5) $$D_{\text{eq}}=1.5\times \left(D_{m\text{ax}}+D_{m\text{in}}\right)-\sqrt{\left(1.5\times D_{m\text{ax}}+0.5\times {\;D}_{m\text{in}}\right)\left(0.5\times D_{m\text{ax}}+1.5\times {\;D}_{m\text{in}}\right)}$$

In (Equations 2, 3, 4, and 5), C is circumference of IVC; a is semi-major axis of the oval; b is semi-minor axis of the oval; D_max_ is maximum diameter, and D_min_ is minimum diameter; D_eq_ is predictive diameter from the predictive model.

#### Measurement of D_max-IVCF_ and D_min-IVCF_, and model validation

Following IVCF placement, postoperative DSA were performed to obtain the IVC maximum diameter (D_max-IVCF_) and minimum diameter (D_min-IVCF_). The ROI for measurement was defined between the superior margin (X_1_) and the inferior margin of the IVCF strut junction (X_2_). The mean D_max-IVCF_ and D_min-IVCF_ were calculated using following formula (Equation 6):

(Equation 6) $$D_{\max-\text{IVCF}}\;{\text{or}\;D}_{\min-\text{IVCF}}=\frac{X_{1}+X_{2}}{2}$$

where D_max-IVCF_ or D_min-IVCF_ is the mean maximum or minimum diameter after IVCF placement; X_1_ is the superior margin of IVCF strut junction, and X_2_ is the inferior margin.

#### Model validation in patient samples

At our hospital, iodinated contrast (Iopromide, Bayer HealthCare) was administered at a rate of 4 mL/s via an 18-gauge cubital intravenous access. CTV imaging was performed using a 128-slice dual-source CT scanner (SOMATOM Definition Flash, Siemens, Germany) during a single inspiratory breath-hold.^16^ Manual measurements of the IVC diameter were performed for each patient, the diameters were measured and recorded from the center of the lumen via the minimum (D_min-patient_) and maximum (D_max-patient_) axes on cross-sectional images.^5^ The predictive diameter (D_eq-patient_) was subsequently calculated using the established Equation 5. For patients with IVCF placement, the post-procedural measurements were taken from the middle edge of one supporting strut to the middle edge of the opposite supporting strut, and recorded as minimum (D_min-IVCF-patient_) and maximum (D_max-IVCF-patient_) diameters.^5^^,^^8^ Two independent reviewers, blinded to clinical data, performed these measurements using CTV images.

### Quantification and statistical analysis

Data were analyzed using SPSS software (version 22.0; SPSS, Chicago, Illinois, USA) and R software (version 4.2.3; R Foundation for Statistical Computing, Vienna, Austria). Continuous variables with a normal distribution were presented as mean ± standard deviation (SD) and compared using Student’s t test. Cloud-rain plots of D_eq_, D_max-IVCF_ and D_min-IVCF_ were generated using GraphPad Prism (version 9.0; GraphPad Software Inc, CA, USA). Lin’s concordance correlation coefficient (CCC) with 95% confidence intervals (CI) was calculated using MedCalc software (version 22.009, Mariakerke, Belgium) to evaluate the agreement among D_max, D_min,__ D_max-IVCF_ and D_min-IVCF_, as well as their correlation with the D_eq_. Moreover, the associations between the D_eq-patient_ and the D_max-IVCF-patient_ or D_min-IVCF-patient_ were calculated. Agreements were classified as poor (CCC ≤0.40), fair to good (0.40 < CCC ≤0.75), or excellent (CCC >0.75).^15^ Differences between CCC were compared using Fisher’s *z*-transformation in MedCalc. A two-sided *p*-value <0.05 was considered statistically significant.
