# Supplementary material for: Development and tracking of inferior vena cava diameter after filter placement using a circle-fitting predictive model
Source: iScience. 2025 Sep 21;28(10):113616. doi: 10.1016/j.isci.2025.113616 (PMC12519178; doi:10.1016/j.isci.2025.113616)
Supplement: Document S1. Figure S1 [file mmc1.pdf]

iScience, Volume *28*

Supplemental information

Development and tracking of inferior vena cava diameter after filter placement using a circle-fitting predictive model

Maofeng Gong, Rui Jiang, Xu He, and Jianping Gu

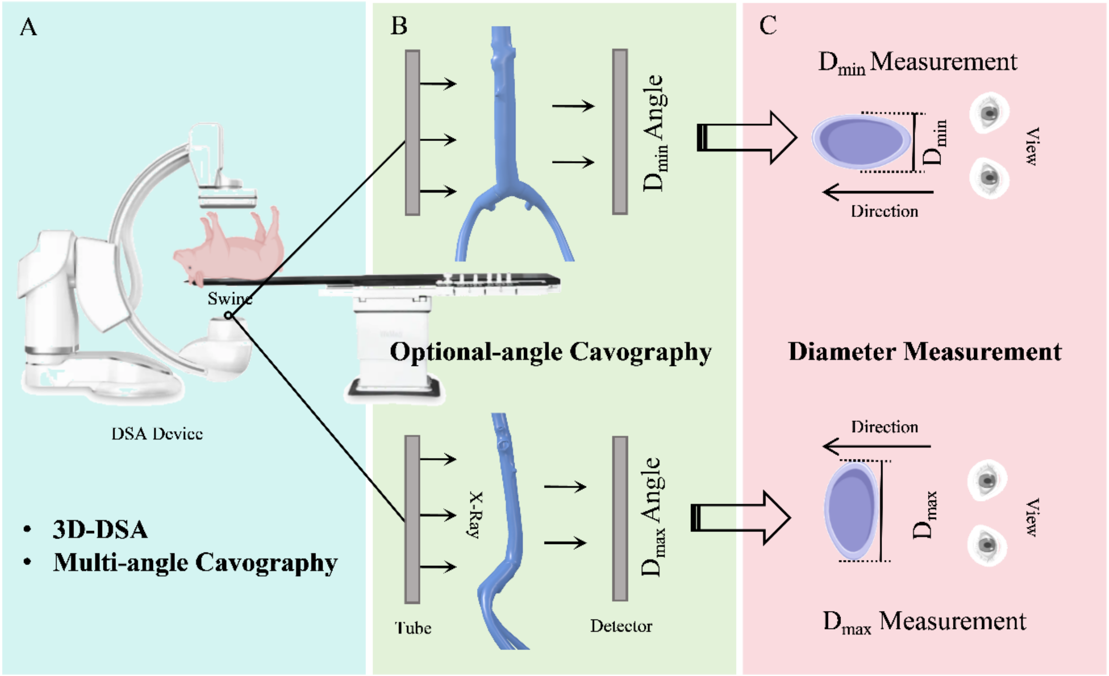

Figure S1: A schematic illustration of this imagine strategy. Related to METHOD DETAILS. A.) Accurate assessment of the inferior vena cava diameter was achieved using 3D DSA reconstructions coupled with multi-angle cavography prior to filter placement. B.) and C.) Optimal imaging angles were selected to enable precise measurement of the D_{min} and D_{max}. Abbreviations: 3D = three-dimensional; DSA = digital subtraction angiography; D_{max} = maximum diameter before filter placement; D_{min} = minimum diameter before filter placement.
